# Operability, Usefulness, and Task-Technology Fit of an mHealth App for Delivering Primary Health Care Services by Community Health Workers in Underserved Areas of Pakistan and Afghanistan: Qualitative Study

**DOI:** 10.2196/18414

**Published:** 2020-09-17

**Authors:** Shehla Zaidi, Abdul Momin Kazi, Atif Riaz, Ammarah Ali, Rabia Najmi, Rawshan Jabeen, Umerdad Khudadad, Saleem Sayani

**Affiliations:** 1 Department of Community Health Sciences Aga Khan University Karachi Pakistan; 2 Department of Paediatrics and Child Health Aga Khan University Karachi Pakistan; 3 Digital Health Resource Centre Aga Khan Development Network Karachi Pakistan

**Keywords:** mHealth, community health workers, usability, usefulness, task-technology fit

## Abstract

**Background:**

The recent proliferation of digital health technology in low- and middle-income countries has made it possible for community health workers (CHWs) to use mobile health (mHealth) to perform tasks such as data collection and training. Although most studies focus on the prospect of digital apps to motivate and connect CHW, only a few have captured end-user experiences with mobile-based apps. We examined the experience of frontline health workers with a move towards digitalized real-time data to record maternal and childcare services in remote areas of Afghanistan and Pakistan.

**Objective:**

Our study aimed to explore CHW perceptions on the operability of the mHealth app in a community setting, usefulness of the app in the delivery of assigned maternal and childcare functions, and the task-technology fit with monitoring information systems.

**Methods:**

The Hayat app, designed to digitalize and facilitate electronic record keeping, was evaluated to be embedded into mainstream health systems. The app had 2 components: smartphone app for data entry and web dashboard for visualization of the maternal, newborn, and child health reports. Using a qualitative exploratory study design, we conducted a total of 8 focus group discussions with purposively selected lady health workers (LHWs) and CHWs in 3 districts of Pakistan and 3 hamlets of Afghanistan, respectively. Focus group discussions were conducted in the local language, audio recorded, and converted into expanded notes for thematic analysis.

**Results:**

Although a majority of LHWs used the app with ease, some initially faced difficulties in operating it and requested a longer duration of training. Contrary to LHWs, the CHWs were able to use the app without difficulty, as they were using it only to register clients. Overall, use of the mHealth app in both countries resulted in a positive impact on health education sessions, easier communication with parents or clients, tracking of routine immunization defaulters and follow-ups, improved data validity, easily accessible vaccination schedules, and faster registration. In addition to building up their image in the community and personal development, the improved reporting and monitoring mechanisms also set the stage for the LHWs to get recognized for their hard work. CHWs in Afghanistan also reported the app provided immediate access to information when requested by their supervisor. Although the Hayat app eliminates the need to carry multiple registers and helps in recalling client information at the touch of a button, technical issues around connectivity and data inputting tabs were highlighted by the participants.

**Conclusions:**

The digitization of records not only provided CHWs support in their daily routine but also strengthened monitoring mechanisms and improved motivation. We recommend conducting end user experience studies before embedding apps into mainstream health systems as high acceptability does not always result in high uptake of digital technology.

## Introduction

The vital role of community health workers (CHWs) as a bridge between the community and health care services is getting renewed attention in low- and middle-income countries (LMICs). CHWs have collectively helped in reducing the maternal and child mortality burden and assisted in decreasing the burden and costs of tuberculosis and malaria [1-3]. Pakistan and Afghanistan have well-structured CHW programs known as the lady health worker (LHW) program in Pakistan and CHW program in Afghanistan, which have provided primary health care services for more than a decade [4]. In both programs, CHWs perform similar functions ranging from health education and awareness; diagnosis and treatment of prevalent diseases such as diarrhea, malaria, respiratory illnesses, and intestinal worms; directly observed therapy for tuberculosis; either antenatal or postnatal health care; and referrals to the health care facilities [5,6]. As Pakistan and Afghanistan still face some of the highest maternal and child mortality burdens and fertility rates in the region [7], the role of frontline health workers is critical to meet the sustainable development goal targets. A supportive work environment, motivation, and competency building are the key factors identified by the framework for strengthening CHW performance [8,9]. However, LHWs often work under little supervision and outreach support in Pakistan [10,11]. Similarly, in Afghanistan, capacity building and establishing support systems have been reported to improve service delivery by CHWs [12].

Cellphone penetration >90% in LMICs, coupled with falling call prices and increased network connectivity options, has improved the feasibility of mobile health (mHealth) programs [13] in remote areas of LMICs, creating the possibility for strengthening weak health systems. CHWs around the globe are using mHealth technology for data collection, training, communication, mobile job aids, decision support tools, and behavior change communication in the community. The few examples of successful mHealth-based facilitation of service provision by CHWs in remote and hard-to-reach areas in LMICs include an increase in the registration and uptake of family planning services in rural Tanzania [14] and an 85% reduction in the average number of days for overdue visits in Dar es Salam, Tanzania [15]. In Pakistan, an app called “e-Vaccs” was introduced in the province of Punjab to track the movement of vaccinators using GPS. The app was implemented to improve vertical accountability through digitalization of records and was supported by the government of Punjab (Punjab Information Technology Board) [16]. This led the way for other provinces to adopt digital immunization solutions to improve vaccination coverage, a prime example being the introduction of the Teeko app in the rural districts of the province of Sindh [13].

The majority of mHealth studies have focused on the prospect of a digital app to motivate and connect CHWs among themselves, their supervisors, and around the facilities [17]. However, few studies have captured end-user experiences with a mobile-based app [18,19]. User experience takes a broader look at the individuals’ entire interaction with the app that includes thoughts, feelings, and perceptions resulting from that interaction [20]. If the intended aim of the app is user satisfaction and improved work efficiency, the understanding of end-user experiences can provide valuable details on the features and functions most needed for the intended task, make available detailed knowledge of issues to be addressed, and offer an experience comparable with the traditional methods of working [21]. Concerns of not having enough data regarding the implementation and evaluation of the app before recommending the embedding of the app into mainstream health systems have been highlighted by academics globally. They fear a graveyard of poor-quality, unproven apps contributing to a fragmented mHealth landscape [22]. Some experts recommend assessing the barriers and challenges faced by the targeted end users before designing the app to be able to provide mHealth as a good-fit solution [20,23]. A study attributed the failure of technology to its lack of regard for user requirements and experience [24]. Another recent mHealth study aiming to inform how digital technology can be embedded within district health systems in LMICs highlighted the need for co-option by end users and district stakeholders. It also reported that ease of operability, satisfaction with reliable data, personal recognition, links to field support, and empowerment are powerful enablers of the shift towards digitalization [13].The purpose of our study was to explore the necessary facets of the initial version of the Hayat app revolving around user centeredness to provide an evidence base informed by the understanding of experiences and needs of CHWs in remote areas of Pakistan and Afghanistan [25].

In this paper, we examine the end-user experiences of CHWs in Afghanistan and LHWs in Pakistan with moving towards digitalized real-time data for recording maternal and childcare services provided in the community. Evidence is drawn from experiences with implementing the Hayaat app, a mobile-based app for CHWs piloted in select remote areas of Pakistan and Afghanistan to track community-based health care delivery. The app was launched with an end goal of scalability throughout the regions. As compared with apps previously introduced in Pakistan, the Hayat app had a scaled-up immunization component from the Teeko app as well as a pilot maternal care component. The theory of change underpinning and guiding this intervention assumes that digital intervention will improve the validity and timeliness of reporting by health workers, allowing for accountability of CHW and LHW performance and on-site support resulting in improved visit frequency and quality. This improved visit frequency and quality are theorized to translate into increased household awareness and practices regarding maternal, newborn, and child health (MNCH) leading to improved health outcomes. Key features of the app included GPS to track CHWs during outreach visits, registering clients in the health facility and during outreach, and MNCH data collection. We draw on the experiences of frontline primary health workers using the mHealth app to inform the shift towards digitalization. The objectives of this study were to explore the perceptions of CHWs on the operability of the app in the community setting, usefulness of the app in the delivery of assigned MNCH functions, and task-technology fit with CHW’s monitoring information systems. We aimed to inform the embedding of the digital app into the mainstream health systems of both countries. We also hoped the findings of this evaluation could contribute to app development in other LMICs and inform the process of end-user evaluations.

## Methods

### Study Setting

This study was nested in a larger ongoing quasiexperimental study assessing the effectiveness of the Hayat app for improving maternal and child health in Pakistan and Afghanistan. In Pakistan, the app was implemented in 9 health care facility catchment areas of the Chitral District in Khyber Pakhtunkhwa Province and in the catchment areas of the districts Astore and Ghizer in the Gilgit-Baltistan Province covering a total population of 310,012 people, of which 49% are women. For this study, 4 union councils (UCs), 2 each from Khyber Pakhtunkhwa and Gilgit-Baltistan, were randomly selected from a list of UCs in which the Hayat app was implemented.

In Afghanistan, the Hayat app was implemented in 4 health facility catchment areas (Iragh, Shunbul, Khandaq, and Kalo) in Bamyan Province and 3 catchment areas (Ghaaran, Baharak, and Ishkashim) in Badakshan Province (total population of 117,878, 49% women). The health facilities in the catchment areas of these 2 provinces are operated by Aga Khan Health Services Afghanistan in collaboration with the Ministry of Public Health. For this study, we selected all the catchment areas from the 2 provinces in which the Hayat app was implemented (Table 1).

**Table 1:** Districts in which the Hayat ap was implemented.

Country	Catchment areas in which the Hayat app was implemented	Target population	Selected areas for this study
Pakistan	Kosht, Karimabad, Mulkhow, Charun, Lotkoh, Shoghore, Chatorkhand, Ishkoman, Bubar, Hatoon, Sherqill, Singal, Gupis, Phander, Pingal, Sumal, Teru, Hundur Silgan, Taus, Thoi, Yasin, Gahkuch	310,012	Buni, Garam Chashma, Gahkuch, Gupis
Afghanistan	Ishkashem, Baharak, Gharaan, Iragh, Shunbul, Khandaq, Kalo	117,878	Ishkashem, Baharak, Gharaan, Kalo, Shunbul, Khandaq, Iragh

### mHealth Intervention (Hayat App)

**Figure 1:**
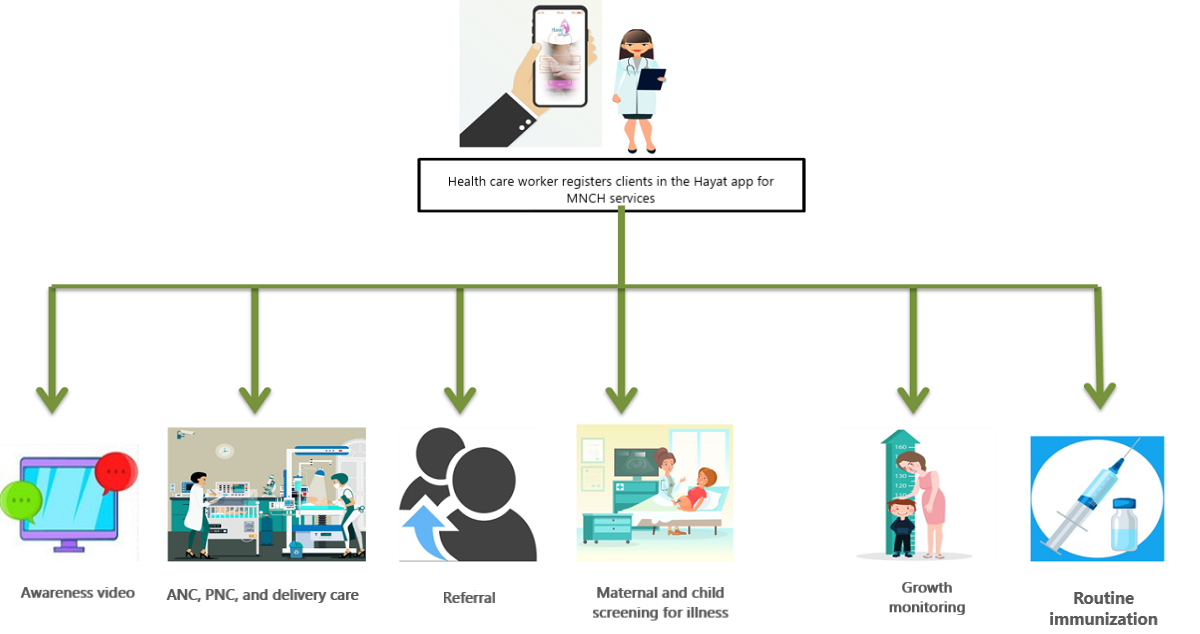
Workflow of the Hayat app for community health workers. ANC: antenatal care; MNCH: maternal, newborn, and child health; PNC: postnatal care.

**Figure 2:**
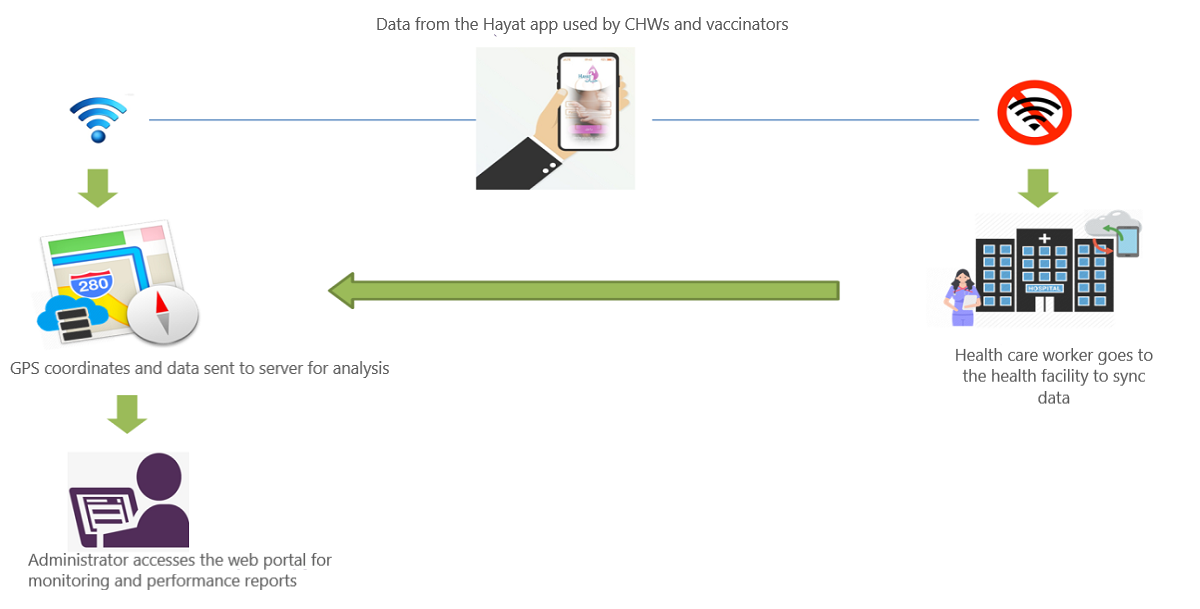
Workflow of the Hayat app for health managers. CHW: community health worker.

The Hayat app was designed to digitalize record keeping, to make it easy for CHWs and shift from paper-based to mobile-based electronic record keeping. The app had two components: smartphone app for data entry and dashboard (web portal) for visualization of the MNCH reports at a later stage. In both countries, the CHWs were provided smartphones with the Hayat app. The app was designed according to the contextual role and scope of the CHWs in the respective country. For further details on the workflow of the Hayat app, refer to Figures 1 and 2.

The Hayat app features include client registration, data collection, GPS, capabilities, and awareness content.

#### Client Registration

Clients can be registered, with their demographic profile, by CHWs in 2 categories: >2 years of age and <2 years of age. To find the client record, the app used multiple means such as fingerprint, QR card, ID card number, or household number.

#### Data Collection

LHWs can record the demographic and health-related data of pregnant women, women of childbearing age, and children <2 years old. Along with the demographic profile of each registered client, data can be recorded for antenatal care (ANC), delivery, postnatal care (PNC), family planning, illnesses, and maternal and child vaccinations.

#### GPS

GPS capabilities allowed real-time tracking of CHWs during outreach visits.

#### Awareness Content

**Figure 3:**
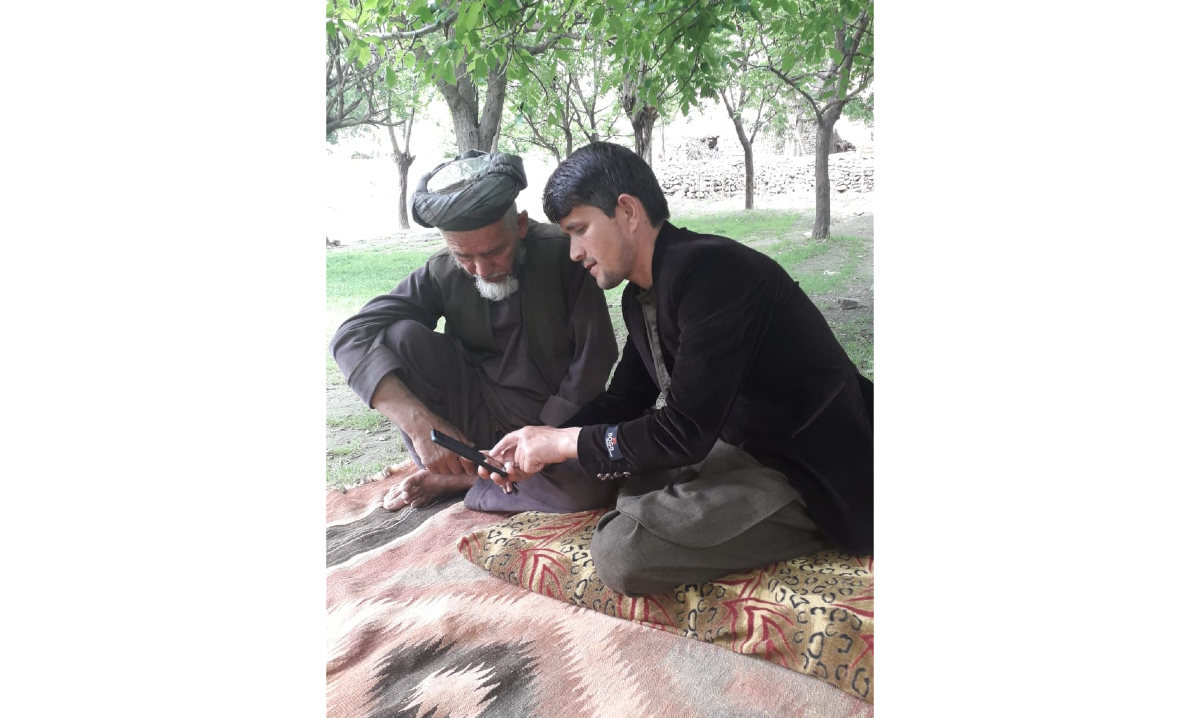
Awareness content being shown to the community.

The app has awareness videos to facilitate the CHWs in health education sessions and outreach activities (Figure 3).

### Framework

We used an adapted framework derived from the task-technology fit model [26] and technology acceptance model [27] to explore the operability, usefulness, and task-technology fit of the mHealth app centered on the end users’ experiences. For this evaluation, we defined the assessment areas as follows.

Operability was defined as the perceptions and barriers such as ease of use, technical competency, and accessibility. This measure aimed to understand the characteristics and capabilities of end users to capture maternal and child health indicators in a resource-constrained setting using digital app.

Usefulness was defined as the perceived satisfaction of health managers and health workers in achieving their goals, including the results and consequences of use.

The task-technology fit of the app was defined as the capability of the app to facilitate the designated tasks of the end users including how the technology interacted with the management information system interface.

In line with the purpose of our study, we used a deductive form of analysis using 3 broad a priori themes of operability, usefulness, and task-technology fit. These themes guided the construction of interview guides, as our goal was to explore these areas in detail to assess whether the app works for our intended users (ie, CHWs) and help tease out any issues with the app.

### Data Collection

Data were collected in July and August 2019 from selected districts of Afghanistan and Pakistan in which the Hayat app was introduced in April 2019. We used a qualitative exploratory study design and conducted focus group discussions (FGDs) with LHWs and CHWs in Pakistan and Afghanistan, respectively. FGD is a widely used tool for collecting data on the perceptions and experiences of health care workers and provides an opportunity for in-depth probing [25]. The moderator started out by asking the participants questions related to their own thoughts and experiences to establish rapport and hear the extent to which our a priori topics were already part of the participants’ thinking before we cued them to think along those terms. Our intention was to provide the necessary user-centered feedback to inform the development cycle of the app for future use, easy embedding into mainstream health systems, and ensuring its sustainability.

A total of 8 FGDs were conducted, including 4 FGDs with LHWs in Pakistan and 4 FGDs with CHWs in Afghanistan, achieving theoretical saturation.

### Selection of Study Participants

#### Pakistan

The participants for this study were purposively selected and approached for FGDs. One FGD was conducted in each selected UC with 10-12 participants each.

#### Afghanistan

Altogether, 4 FGDs were conducted with 8-12 purposively selected participants in each session: 2 in Shebar district, 1 in Baharak district, and 1 in Ishkesham district. The sample size and participant characteristics are provided in Table 2.

**Table 2:** Characteristics of the community health workers (CHWs) and lady health workers (LHWs).

Characteristics		Pakistan (n=54)	Afghanistan (n=42)
Age (years), mean (SD)		39 (5.7)	39 (13.3)
**Level of education, n (%)**			
	<10 years	0	25 (60)
	At least matriculation (10-14 years)	34 (63)	14 (33)
	Graduate (≥16 years)	20 (37)	3 (7)
Years of service, mean (SD)		18.6 (7.1)	10 (5.7)

A semistructured interview guide was developed for the FGDs for both countries, using relevant literature and adapted to the local context. Questions were framed around the a priori identified themes for the mobile app including operability, perceived usefulness, and task-technology fit. Probes were added where necessary to steer the discussions. Guides were developed in the English language and translated into local languages (ie, Urdu for Pakistan and Dari for Afghanistan). Experienced data collectors were hired and trained to conduct FGDs, manage data, and maintain professional and ethical conduct throughout the data collection. A free flow of discussion was ensured during the FGDs, and all participants were encouraged to contribute to the discussion. Notes were taken by the interviewer during the interviews and FGDs. The main points were summarized by the interviewer at the end of each interview and FGD to get confirmation from the participants. The interviews were conducted by one researcher in each country. These researchers belonged to the country assigned and were proficient in the local language. This ensured cultural sensitivity and familiarity with the area. The FGDs were arranged at a time and place convenient for participants, and each FGD lasted 40-50 minutes. To limit bias, the research team that undertook tool development, collection, and analyses was not part of the intervention team.

The interviews and FGDs were recorded using a digital recorder. All electronic data were stored on encrypted computers, and consent forms and recordings were stored in a locked drawer in the Community Health Sciences department at Aga Khan University.

### Data Analysis

Recordings were transcribed verbatim in Urdu and Dari and translated into English at the Community Health Sciences department, Aga Khan University Pakistan campus by the research teams from Pakistan and Afghanistan, respectively. Translations were reviewed for accuracy by experienced researchers who had command of both the local and English languages. Personal identifiers were removed, and unique IDs were assigned to each participant in the FGDs. Transcripts were reviewed repeatedly to gain familiarity with the data. A thematic content analysis of the textual data was conducted using Nvivo 10 by 2 trained researchers from the team. Transcripts from Pakistan and Afghanistan were coded separately, and similar codes were categorized under the aforementioned thematic areas (ie, operability, perceived usefulness, and task-technology fit). Within each thematic area, further deductive coding was conducted based on emerging concepts from the narrative.

### Ethical Considerations

Approval was obtained from the ethical review committee of Aga Khan University Hospital (ERC#2018-0375-951) prior to the start of the study, and institutional review board approval was obtained from the Afghanistan National Public Health Institute (IRB# AIRB-0419-0014). Written informed consent was obtained from all participants after explaining the purpose of the study.

## Results

### Operability

#### Pakistan

Despite the concerns shared by the LHWs regarding the short training period for the app, the majority stated that they were able to use the app with ease. A few mentioned that they faced difficulties in operating the app initially; however, they were able to work with it after a bit of trial and error and ongoing facilitation by the supervisors. Regarding the language of the app, in one of the FGDs, the LHWs suggested that it should be changed to Shina (a local language) as some of them had to translate the app features into Shina for some of the mothers to understand. The LHWs that were unable to translate were helped by other LHWs. It was also noted by the LHWs that the monthly call package helped them in paying the costs of the calls, which provided an efficient way of communicating with mothers and caretakers regarding their child’s health. They were able to connect through cell phones with the mothers and caretakers, reducing extra unnecessary household visits and more efficient use of their time. There was a reluctance to make the calls without a prepaid call package, as was the case during initial days of the app implementation.

At first, we made many mistakes such as writing a female name in the male section. It was because we were not experienced at all. It took time but we learned.

LHW Booni

Now, we don't have to go everywhere. We get a phone package, and we call them [mothers]. Earlier, we used to call them using our own phones.

LHW Gahkuch-Ghizer

It would be great if the language of the app is changed to Shina.

LHW Booni

#### Afghanistan

The CHWs expressed that the app was user friendly, and they were able to use it without any trouble because the app was being used for registration of clients only and not for documentation of health status. They also mentioned being supported in the outreach by the Aga Khan Health Services Afghanistan personnel that provided technical support in operating the app. The CHWs were operating the app in their local language (ie, Dari).

Some people did not give us the tazkira number, which was a problem. But it is resolved now. We talked to the people who gave us this mobile phone about this, and they have asked us to just put 0799 in place of the tazkira number. This is not a problem now.

CHW Baharak

### Usefulness

#### Pakistan

The majority of the LHWs agreed that the awareness videos for the community outreach education sessions were very useful. However, they pointed out that additional videos on other topics such as depression and cancer are also needed. According to one LHW, to change the behavior of the community, variation in the methods of health education sessions is needed. Furthermore, some of the LHWs noted an increase in the communities’ response to the follow-up on vaccination and reported that the community viewed the videos with interest and asked questions regarding child health.

They (videos) are beneficial for mothers because the videos attract them, and they watch them with interest.

LHW Garam Chasma

We give health education in health care centers, schools, and mosques too, so if we have videos of other diseases, that would be great.

LHW Booni

What we used to tell them verbally wasn’t that effective. Now, conducting sessions with videos makes it really interesting, and they understand better.

LHW Gupis

The digitalization of health records improved the work efficiency of the LHWs, as they were able to complete an hour’s worth of tasks in just a few minutes. Most of the LHWs reported that digitalization made the procedure of vaccination easier as, instead of entering data in multiple registers, LHWs can enter information in a single mobile device. They are also able to contact mothers for the scheduled vaccines; previously, they did not have enough calling credits. The LHWs noted that the app showed not only defaulter children by changing the colors of the entry but also the vaccination schedule of each child, which helped them list and contact mothers. The app was also reported to be helpful for monitoring child growth. The LHWs shared that, before the app, they did not weigh the children; however, with the app, they are required to weigh them for monitoring growth. The LHWs claimed they did not have growth charts for the last 3-4 years. However, now they have a growth chart in the app, which they can show mothers to inform them about their child’s nutrition and health status

It has made our work easier now. Before, for vaccination, we had to do entry in different registers, but now we only need to enter in the mobile.

LHW Garam Chasma

This [growth chart] is also useful for the mother, as they get to know about the health of the children; if they are skinny, they concentrate on the lines because they don't want their children to be skinny.

LHW Gahkuch Ghizer

The LHWs shared that the app had a positive effect in building up their image in the community. They felt that, with the introduction of the app, the community now trusted them and showed respect. The LHWs were pleased about the possibility of getting recognized for their hard work. They shared that the department was unhappy with their collective work and called them out for it despite their efforts, even during the campaigns. They hoped that the digitalization of records would allow for individual performance monitoring and give them their due credit. The LHWs also shared their changed attitude towards their work. Before the app, they had grown tired of working with multiple heavy registers for a long time. The replacement of these registers by a mobile phone brought excitement into their work and motivated them. Many stated that this was their first mobile phone and reported developing skills in using it and considered it as a skill-building opportunity. They reported improved self-esteem and confidence.

Our department is not happy with us. They criticize us for not working. We are lucky that we have mobile phones now so that we can get credit for all the work we do. We are very happy with this app.

LHW Booni

We work very passionately. Because of the mobile phone, we have changed. Before, we didn't know how to use a mobile phone, but we got it because of this project.

LHW Gahkuch Ghizer

We are very happy with mobile; it has made our work very easy. We enjoy our work now. We are happy.

LHW Booni

#### Afghanistan

The CHWs shared that they were most excited about using the video feature in the app for conducting health sessions at different locations in the community. This facilitated them in addressing communities’ concerns and raising awareness regarding the side effects of vaccination. The CHWs noted these videos to be beneficial for they created awareness and harnessed the community’s interest.

When we just speak, it doesn’t have a big impact. People remember things by watching. The good thing about this app is the videos. People can see it as well as listen.

CHW Ishkashim

Once a month, there is a meeting where all the community people are invited — sometimes in the mosque or at the home of community leaders. We have the responsibility of health education, so we use these videos to educate them about immunization, and they become more motivated for vaccination after watching this.

CHW Baharak

Now people have become aware. They ask us to give them information not just on vaccination but also about nutrition, tuberculosis, and diabetes…every kind of disease.

CHW Baharak

The CHWs reported increased efficiency of their work due to the digital app. They were able to comparatively register more children for vaccination and report their information to the vaccinators in a timely manner so that they can complete vaccinations. They also were able to share the child vaccination status with the parents and connect them to a nearby health care facility. The CHWs felt that the process of MNCH services was accelerating, as the app helped them focus on their catchment areas only. This helps vaccinators find the exact location of the child for routine immunization. Moreover, the saved information and vaccination schedule allowed them to track defaulter children. The digitalization of records eliminated duplication of work because the data are now saved in the app; earlier, they had to be careful to avoid losing information through torn, missing, and wet pages. It also facilitated the reporting of data to the clinic because they did not have to carry multiple registers.

For vaccination, the parents should be connected with the nearby health facility, which was missing previously. Parents keen to get their children vaccinated didn’t have enough information about the vaccination status of their children and which health facility to go for vaccination. It is resolved through this app.

CHW Shebar

Before, when we came across someone who asked for the vaccination status of their child, it was difficult for us because the register was at home. But now the same register is in our pocket. We can register children on the spot and share any information if they need it.

CHW Baharak

The positive influence of the app on the community was pointed out by the CHWs. In case the outreach team was unable to vaccinate a child, the mother could bring the child to the facility for vaccination. The app also helped the CHWs respond to community questions. The CHWs were pleased about improved reporting mechanisms and having information at hand through the mobile phone when asked by their supervisors. The CHWs reported personal development through the app. The app not only helped them get acquainted with digital technology but also improved their knowledge base regarding MNCH services.

My knowledge about the MNCH services was low before the introduction of this app, and I wasn’t able to respond to some of the mothers’ queries. But now, I have gone through the videos many times, and it has helped me to understand MNCH services such as ANC, PNC, breastfeeding, immunization, and family planning.

CHW-Shebar 2

From the perspective of reporting, it has helped us in a great way. We were unable to give the right report to the health facility before this app. They weren’t even happy with our work. But now, the reporting is good, and we are not losing any data.

FGD 4 Ishkashim

### Task-Technology Fit

Task-technology fit was defined as the capability of the app to facilitate the designated tasks of the end users including interoperability (ie, how the technology interacts with the management information system interface).

#### Pakistan

The LHWs expressed that all the work and data entry they did in multiple registers was now being done in a single mobile phone, eliminating the need to carry multiple heavy registers in the field for data recording. They also used the app to identify defaulter children for routine immunization. The LHWs were happy with the online data synchronization, as it will highlight their hard work for higher management. Nevertheless, the LHWs noted their frustration over not being able to edit or delete the client records. This required them to reenter data, which then created duplicate registrations. One LHW shared that because the baby was not named at the time of registration, she had to register the child again as the app would not allow her to edit the baby’s name. Also, some features present in the LHW register were reported missing in the app. The app did not include the monthly plans made by the LHWs nor the community chart that includes the total population, number of kids, and death and birth records. They also raised concerns regarding the limited options to choose from in certain categories such as nutrition and adverse outcomes in pregnant mothers.

It’s an online system, and it will highlight our hard work to the top.

LHW Booni

There are fewer options than our registers — like if a pregnant woman has a mishap, we enter that in our register, but we can’t enter it in the app.

LHW Gahkuch Ghizer

#### Afghanistan

The CHWs expressed appreciation for being able to recall client information at the touch of a button. Earlier, this level of recall was only possible by going through multiple registers. They also felt that the data are now more secure in the central server than in the registers. However, the CHWs voiced their apprehensions regarding the lack of connectivity and synchronization of data with the central server located at the Aga Khan Health Service office. They had to travel long distances every week for data transfer. The CHWs pointed to the lack of network coverage in their regions as the major hindrance to data transfer, as it requires internet connectivity. Their coordination with the health workers in facilities was also affected as they were not able to inform them of their referred cases in time. They also raised concerns regarding the lack of editing and deleting options in the app. This was a problem, as this did not allow them to edit mistakes and information. The CHWs also wanted to have the option to generate a list of children according to their gender.

This app is an ever-ready resource for us. Anytime we want something — information on the vaccine status of child or anything else — we can easily find it.

CHW Ishkashim

The negative thing about this app is that the data are not synchronized with the portal system, and we have to travel a distance of 3 hours every week to transfer the data into the portal manually.

CHW Shebar

## Discussion

### Principal Findings

Although the importance of including end users’ perspective for effective implementation of mHealth interventions has been emphasized in the past [28], the contribution of qualitative evaluation of mHealth apps to supplement their implementation has not received much attention in most South Asian countries. This study adds important insights on the function and applicability of the Hayat mHealth app in facilitating routine work of LHW and CHWs in Pakistan and Afghanistan, respectively.

Our findings show that most of the LHWs and CHWs found the app easy to use and it facilitated day-to-day tasks in Pakistan and Afghanistan. Table 2 compares the characteristics of the sampled participants. While the mean age of participants was 39 years in both countries, the level of education and years of experience were higher in participants from Pakistan compared with participants in Afghanistan. The reason for these differences is that LHWs in Pakistan are paid government employees whereas CHWs in Afghanistan work as volunteers. Furthermore, the LHWs are required to have completed at least matriculation, whereas there is no such requirement for recruitment of CHWs. Even though the 2 populations differ significantly, the findings related to the operability and usefulness of the app were largely similar in both countries. However, the app was being used for the full range of MNCH services in Pakistan in contrast to Afghanistan, where its use was limited to client registration, education, referral, and immunization. The app was found to be useful on many fronts; some of the encouraging results highlighted in this study included improvement of service delivery planning, time management and efficiency, accuracy of collected data, communication with caregivers, caregiver’s compliance with instructions, individual LHW performance accountability, and simplification of the work routine. CHWs in Uganda and Mozambique also felt that an mHealth app had the potential to improve their work efficiency, planning, and communication with supervisors [29]. Offline features of the app and awareness videos were reported to be most useful when conducting outreach visits, supporting the use of videorecorded health messages for raising awareness in communities. Findings reported from a study conducted in Pakistan indicated the usefulness of an offline feature; however, awareness videos on routine immunization were reported as a less-used feature by the vaccinators [13].

Simultaneously, findings of this study also highlighted the challenges faced by end users in remote and hard-to-reach areas that will require addressing to maximize the utilization of the app in resource-constrained settings. First, network coverage was limited, and while the offline feature prevented data from being lost, the users still felt frustration at not being able to sync data in real-time as it then became a pending or additional task requiring their attention later. In addition, issues in communicating referrals with health facilities and traveling long distances to upload data were some of the other constraints. Second, the capacity building of digital literacy skills for those in need should be carried out on a regular basis to maximize the output of an mHealth intervention. Third, because the CHWs were fully aware of the capabilities of the dashboard and app to monitor individual performance, there was a strong sense that acknowledgment of individual performance was expected. They were not satisfied with the current supervision mechanisms, which masked individual efforts. They were judged on cumulative performance, without individual acknowledgment, creating grounds for favoritism and unjust admonishment. The study also highlighted that the CHWs hoped that the app would help address the need to constantly prove their efforts to their supervisors and dedication to the program, especially in Afghanistan, where they are volunteers [6].

Our study showed that management of data was facilitated, reducing missing and erroneous entries and improving transparency and accountability. Multiple ways of registration allowed for many ways to recall data if one form of information was lost. The CHWs were also excited about the possibility of abolishing registers that were a hassle to work with. CHWs using the Geohealth app in Brazil also reported that replacing paper with the app greatly reduced the load they carried into the field [30].

Technical issues highlighted during the study need to be considered because they strongly contribute to the usability of the app; if not corrected, these issues ultimately threaten uptake. A study in São Paulo, Brazil highlighted that the technical characteristics of an app should align with the task features of CHWs for it to be operable, useful, and sustainable. It also highlighted the need to involve end users in assessing the technical barriers in hardware and software to maximize the utilization of an mHealth app [30].

The benefits of equipping health workers with data management technologies to improve the continuity of care in hard-to-reach populations have been established in the existing literature. Global examples revealed high levels of acceptance and willingness to learn despite lack of experience with such tools [31-33]. It also shows that the use of mHealth apps by CHWs has improved outreach services, data collection and management, and outreach activities [34,35]. Moreover, a digital app has the potential to improve users’ knowledge, skills, and performance [36-38]. The literature shows that the contribution of an app to the improvement of health outcomes depends on its perceived usefulness and usability by the users. It highlights the role of the users in the successful implementation of an mHealth app [39,40]. The usefulness of the mHealth app for CHWs is centered on the ease of integration into the routine workflow, improved capacity to deliver follow-up services, less paperwork, organization of data, and abolishing the paper-based registers [30-41]. Moreover, a boost in the CHWs motivation when provided with mobile phones was reported in a study conducted by Madon et al [37].

### Strengths and Limitations

Iterative qualitative methods used to explore the experiences of the end users helped identify factors influencing the uptake of the digital app.

The participants’ responses may have been skewed towards being socially acceptable, hence creating a social desirability bias. Because this study involved cell phones (desired product), the bias might be significant. Several strategies were adopted to avoid or limit any social desirability bias. The FGD participants were volunteers, and the FGDs were conducted at a time and place convenient to the participants to make them feel comfortable [42]. Interviewees were only provided a brief overview of the study at the beginning to avoid preparing respondents to answer in a socially acceptable way [43]. Moreover, the interviews were conducted by an experienced moderator that strived to create an open and comfortable atmosphere for the participants to share their viewpoints and experiences [44]. The participants were assured that there was no incorrect answer, their responses would not affect their participation or lead to their mobile phones (given as part of the study) being taken away, and the FGD was about hearing their diverse views and understanding their feelings. They were also encouraged to support their views with personal experiences [45]. These strategies collectively provided confidence that any social desirability bias was reduced.

### Conclusions

Involving end users is necessary for successful integration of an mHealth app into existing programs. Qualitatively evaluating the digital app from the lens of operability, usefulness, and task-technology fit provided us with a broader picture of the factors that could affect its uptake in the longer term. Our findings indicate that the app being in the local language, supplemented with a simple interface, made it easy for the CHWs to adopt and use it. It saved time, organized the work routine, removed the need to work with manual data registers, and promoted accountability. The relevance of this study extends beyond the 2 countries to similar LMIC settings.

## Abbreviations

ANC: antenatal care
CHW: community health worker
FGD: focus group discussion
LHW: lady health worker
LMIC: low- and middle-income country
mHealth: mobile health
MNCH: maternal, newborn, and child health
PNC: postnatal care
UC: union council

## References

[1] Christopher JB, Le May A, Lewin S, Ross DA (2011). Thirty years after Alma-Ata: a systematic review of the impact of community health workers delivering curative interventions against malaria, pneumonia and diarrhoea on child mortality and morbidity in sub-Saharan Africa. Hum Resour Health.

[2] Langston A, Weiss J, Landegger J, Pullum T, Morrow M, Kabadege M, Mugeni C, Sarriot E (2014). Plausible role for CHW peer support groups in increasing care-seeking in an integrated community case management project in Rwanda: a mixed methods evaluation. Glob Health Sci Pract.

[3] Scott K, Beckham SW, Gross M, Pariyo G, Rao KD, Cometto G, Perry HB (2018). What do we know about community-based health worker programs? A systematic review of existing reviews on community health workers. Hum Resour Health.

[4] Najafizada SAM, Labonté R, Bourgeault IL (2019). HRH dimensions of community health workers: a case study of rural Afghanistan. Hum Resour Health.

[5] Folz R, Ali M (2018). Task sharing in health workforce: An overview of community health worker programmes in Afghanistan, Egypt and Pakistan. East Mediterr Health J.

[6] Najafizada SAM, Labonté R, Bourgeault IL (2014). Community health workers of Afghanistan: a qualitative study of a national program. Confl Health.

[7] Akseer N, Kamali M, Arifeen SE, Malik A, Bhatti Z, Thacker N, Maksey M, D'Silva H, da Silva IC, Bhutta ZA (2017). Progress in maternal and child health: how has South Asia fared?. BMJ.

[8] (2010). Community systems strengthening framework.

[9] Jaskiewicz W, Tulenko K (2012). Increasing community health worker productivity and effectiveness: a review of the influence of the work environment. Hum Resour Health.

[10] Lehmann U, Sanders D (2007). Community health workers: what do we know about them. The state of the evidence on programmes, activities, costs and impact on health outcomes of using community health workers Geneva: World Health Organization.

[11] Rabbani F, Shipton L, Aftab W, Sangrasi K, Perveen S, Zahidie A (2016). Inspiring health worker motivation with supportive supervision: a survey of lady health supervisor motivating factors in rural Pakistan. BMC Health Serv Res.

[12] Edward A, Branchini C, Aitken I, Roach M, Osei-Bonsu K, Arwal SH (2015). Toward universal coverage in Afghanistan: A multi-stakeholder assessment of capacity investments in the community health worker system. Soc Sci Med.

[13] Zaidi S, Shaikh SA, Sayani S, Kazi AM, Khoja A, Hussain SS, Najmi R (2020). Operability, Acceptability, and Usefulness of a Mobile App to Track Routine Immunization Performance in Rural Pakistan: Interview Study Among Vaccinators and Key Informants. JMIR Mhealth Uhealth.

[14] (2020). Using Mobile Health Applications to Improve Family Planning Services. The David and Lucile Packard Foundation.

[15] DeRenzi B, Findlater L, Payne J, Birnbaum B, Mangilima J, Parikh T (2012). Improving community health worker performance through automated SMS. https://www.itu.int/en/ITU-D/Statistics/Pages/facts/default.aspx.

[16] Tracking Vaccinators (E-VACCS). Punjab Information Technology Board, Government of the Punjab.

[17] Early J, Gonzalez C, Gordon-Dseagu V, Robles-Calderon L (2019). Use of Mobile Health (mHealth) Technologies and Interventions Among Community Health Workers Globally: A Scoping Review. Health Promot Pract.

[18] Braun R, Catalani C, Wimbush J, Israelski D (2013). Community health workers and mobile technology: a systematic review of the literature. PLoS One.

[19] Källander K, Tibenderana JK, Akpogheneta OJ, Strachan DL, Hill Z, ten Asbroek AHA, Conteh L, Kirkwood BR, Meek SR (2013). Mobile health (mHealth) approaches and lessons for increased performance and retention of community health workers in low- and middle-income countries: a review. J Med Internet Res.

[20] Albert W, Tullis T (2013). Measuring the user experience: collecting, analyzing, and presenting usability metrics.

[21] Lavallee DC, Chenok KE, Love RM, Petersen C, Holve E, Segal CD, Franklin PD (2016). Incorporating Patient-Reported Outcomes Into Health Care To Engage Patients And Enhance Care. Health Aff (Millwood).

[22] Roess A (2017). The Promise, Growth, and Reality of Mobile Health - Another Data-free Zone. N Engl J Med.

[23] Kazi AM, Ahsan N, Khan A, Jamal S, Kalimuddin H, Ghulamhussain N, Wajidali Z, Muqeet A, Zaidi F, Subzlani M, McKellin W, Ali A, Collet J (2019). Personalized Text Messages and Automated Calls for Improving Vaccine Coverage Among Children in Pakistan: Protocol for a Community-Based Cluster Randomized Clinical Trial. JMIR Res Protoc.

[24] Schnall R, Rojas M, Bakken S, Brown W, Carballo-Dieguez A, Carry M, Gelaude D, Mosley JP, Travers J (2016). A user-centered model for designing consumer mobile health (mHealth) applications (apps). J Biomed Inform.

[25] O.Nyumba T, Wilson K, Derrick CJ, Mukherjee N (2018). The use of focus group discussion methodology: Insights from two decades of application in conservation. Methods Ecol Evol.

[26] Goodhue DL, Thompson RL (1995). Task-Technology Fit and Individual Performance. MIS Quarterly.

[27] Davis F, Bagozzi R, Warshaw P (1989). User Acceptance of Computer Technology: A Comparison of Two Theoretical Models. Management Science.

[28] Chaiyachati KH, Loveday M, Lorenz S, Lesh N, Larkan L, Cinti S, Friedland GH, Haberer JE (2013). A pilot study of an mHealth application for healthcare workers: poor uptake despite high reported acceptability at a rural South African community-based MDR-TB treatment program. PLoS One.

[29] Thondoo M, Strachan DL, Nakirunda M, Ndima S, Muiambo A, Källander K, Hill Z, InSCALE Study Group (2015). Potential Roles of Mhealth for Community Health Workers: Formative Research With End Users in Uganda and Mozambique. JMIR Mhealth Uhealth.

[30] Schoen J, Mallett JW, Grossman-Kahn R, Brentani A, Kaselitz E, Heisler M (2017). Perspectives and experiences of community health workers in Brazilian primary care centers using m-health tools in home visits with community members. Hum Resour Health.

[31] Medhanyie AA, Little A, Yebyo H, Spigt M, Tadesse K, Blanco R, Dinant G (2015). Health workers' experiences, barriers, preferences and motivating factors in using mHealth forms in Ethiopia. Hum Resour Health.

[32] Sukums F, Mensah N, Mpembeni R, Kaltschmidt J, Haefeli WE, Blank A (2014). Health workers' knowledge of and attitudes towards computer applications in rural African health facilities. Glob Health Action.

[33] Zakane SA, Gustafsson LL, Tomson G, Loukanova S, Sié Ali, Nasiell J, Bastholm-Rahmner P (2014). Guidelines for maternal and neonatal "point of care": needs of and attitudes towards a computerized clinical decision support system in rural Burkina Faso. Int J Med Inform.

[34] Blanas DA, Ndiaye Y, MacFarlane M, Manga I, Siddiqui A, Velez O, Kanter AS, Nichols K, Hennig N (2015). Health worker perceptions of integrating mobile phones into community case management of malaria in Saraya, Senegal. Int Health.

[35] Schuttner L, Sindano N, Theis M, Zue C, Joseph J, Chilengi R, Chi BH, Stringer JSA, Chintu N (2014). A mobile phone-based, community health worker program for referral, follow-up, and service outreach in rural Zambia: outcomes and overview. Telemed J E Health.

[36] Long L, Pariyo G, Kallander K (2018). Digital Technologies for Health Workforce Development in Low- and Middle-Income Countries: A Scoping Review. Glob Health Sci Pract.

[37] Madon S, Amaguru JO, Malecela MN, Michael E (2014). Can mobile phones help control neglected tropical diseases? Experiences from Tanzania. Soc Sci Med.

[38] Mc Kenna P, Babughirana G, Amponsah M, Egoeh SG, Banura E, Kanwagi R, Gray B (2019). Mobile training and support (MOTS) service-using technology to increase Ebola preparedness of remotely-located community health workers (CHWs) in Sierra Leone. Mhealth.

[39] Gagnon M, Ngangue P, Payne-Gagnon J, Desmartis M (2016). m-Health adoption by healthcare professionals: a systematic review. J Am Med Inform Assoc.

[40] Opoku D, Stephani V, Quentin W (2017). A realist review of mobile phone-based health interventions for non-communicable disease management in sub-Saharan Africa. BMC Med.

[41] Rothstein JD, Jennings L, Moorthy A, Yang F, Gee L, Romano K, Hutchful D, Labrique AB, LeFevre AE (2016). Qualitative Assessment of the Feasibility, Usability, and Acceptability of a Mobile Client Data App for Community-Based Maternal, Neonatal, and Child Care in Rural Ghana. Int J Telemed Appl.

[42] Brunk KH (2010). Reputation building: beyond our control? Inferences in consumers' ethical perception formation. Journal of Consumer Behaviour.

[43] Steenkamp L, Venter D, Walsh C, Dana P (2014). Socio-economic and demographic factors related to HIV status in urban informal settlements in the Eastern Cape, South Africa. Afr J AIDS Res.

[44] Hollander J (2016). The Social Contexts of Focus Groups. Journal of Contemporary Ethnography.

[45] Bergen N, Labonté R, Asfaw S, Mamo A, Abebe L, Kiros G, Morankar S (2019). Social Desirability Bias in Qualitative Research: What is it and what can researchers do about it?.

